# Supplementary material for: Hot spot prediction in protein-protein interactions by an ensemble system
Source: BMC Syst Biol. 2018 Dec 31;12(Suppl 9):132. doi: 10.1186/s12918-018-0665-8 (PMC6311905; doi:10.1186/s12918-018-0665-8)
Supplement: Supplementary file 2 — All relevant MATLAB code. A simple Matlab implement of our predictor. (ZIP 19,742 kb) [file 12918_2018_665_MOESM2_ESM.zip › Additional file 2/Read ASA/ASA Property/SKEMPI/1DANU.txt]

 NetSurfP - Protein Surface Accessibility and Secondary Structure Predictions

  

|  |  |  |
| --- | --- | --- |
|  |  | NetSurfP - Protein Surface Accessibility and Secondary Structure Predictions  Technical University of Denmark |

  


---

```
# For publication of results, please cite:
# A generic method for assignment of reliability scores applied to solvent accessibility predictions.
# Bent Petersen, Thomas Nordahl Petersen, Pernille Andersen, Morten Nielsen and Claus Lundegaard
# BMC Structural Biology 2009, 9:51 doi:10.1186/1472-6807-9-51
#
# Column 1: Class assignment - B for buried or E for Exposed - Threshold: 25% exposure, but not based on RSA
# Column 2: Amino acid
# Column 3: Sequence name
# Column 4: Amino acid number
# Column 5: Relative Surface Accessibility - RSA
# Column 6: Absolute Surface Accessibility
# Column 7: Z-fit score for RSA prediction
# Column 8: Probability for Alpha-Helix
# Column 9: Probability for Beta-strand
# Column 10: Probability for Coil
E E  1DANU                  1    0.902 157.667   0.394   0.003   0.003   0.994
E P  1DANU                  2    0.451  64.025  -1.176   0.018   0.019   0.964
E L  1DANU                  3    0.426  78.092  -0.574   0.018   0.047   0.935
B Y  1DANU                  4    0.262  56.032  -0.299   0.052   0.084   0.864
E E  1DANU                  5    0.431  75.348  -0.897   0.056   0.142   0.802
E N  1DANU                  6    0.561  82.116  -0.320   0.064   0.216   0.721
B S  1DANU                  7    0.289  33.824  -0.370   0.018   0.088   0.893
E P  1DANU                  8    0.580  82.373  -0.390   0.018   0.088   0.893
E E  1DANU                  9    0.531  92.696   0.458   0.019   0.141   0.840
B F  1DANU                 10    0.062  12.363  -0.524   0.019   0.141   0.840
E T  1DANU                 11    0.321  44.550   0.383   0.018   0.088   0.893
B P  1DANU                 12    0.184  26.110  -0.309   0.056   0.142   0.802
E Y  1DANU                 13    0.282  60.178  -0.245   0.064   0.216   0.721
E L  1DANU                 14    0.434  79.410   0.380   0.125   0.227   0.648
E E  1DANU                 15    0.468  81.777  -0.499   0.064   0.216   0.721
B T  1DANU                 16    0.132  18.322  -0.820   0.064   0.216   0.721
E N  1DANU                 17    0.374  54.754  -0.148   0.066   0.296   0.638
B L  1DANU                 18    0.111  20.251  -0.607   0.021   0.279   0.699
E G  1DANU                 19    0.294  23.106  -1.064   0.018   0.088   0.893
E Q  1DANU                 20    0.415  74.030   0.166   0.018   0.047   0.935
B P  1DANU                 21    0.216  30.721  -0.683   0.004   0.138   0.858
E T  1DANU                 22    0.433  60.002  -0.045   0.004   0.616   0.381
B I  1DANU                 23    0.085  15.762   0.047   0.001   0.900   0.099
E Q  1DANU                 24    0.419  74.887   0.967   0.001   0.959   0.040
B S  1DANU                 25    0.151  17.650   0.073   0.001   0.959   0.040
E F  1DANU                 26    0.372  74.640   0.555   0.001   0.959   0.040
E E  1DANU                 27    0.434  75.855  -0.107   0.001   0.900   0.099
E Q  1DANU                 28    0.452  80.817  -0.237   0.004   0.616   0.381
E V  1DANU                 29    0.595  91.405  -1.210   0.005   0.262   0.733
E G  1DANU                 30    0.722  56.821  -0.695   0.005   0.045   0.951
E T  1DANU                 31    0.317  43.899   0.072   0.005   0.336   0.660
E K  1DANU                 32    0.354  72.900   1.215   0.001   0.900   0.099
B V  1DANU                 33    0.035   5.380   0.383   0.001   0.959   0.040
B N  1DANU                 34    0.263  38.489   1.439   0.001   0.959   0.040
B V  1DANU                 35    0.023   3.550   1.123   0.001   0.959   0.040
B T  1DANU                 36    0.161  22.289   1.140   0.001   0.959   0.040
B V  1DANU                 37    0.025   3.904   0.634   0.001   0.900   0.099
E E  1DANU                 38    0.357  62.403   0.921   0.003   0.718   0.279
B D  1DANU                 39    0.132  18.978   0.053   0.004   0.197   0.799
B E  1DANU                 40    0.155  27.061  -0.112   0.018   0.019   0.964
E R  1DANU                 41    0.510 116.790  -0.580   0.018   0.047   0.935
E T  1DANU                 42    0.345  47.851  -0.727   0.052   0.084   0.864
E L  1DANU                 43    0.249  45.629  -1.596   0.052   0.084   0.864
B V  1DANU                 44    0.235  36.150  -0.873   0.056   0.142   0.802
E R  1DANU                 45    0.429  98.149  -0.345   0.064   0.216   0.721
E R  1DANU                 46    0.610 139.667  -0.809   0.056   0.142   0.802
E N  1DANU                 47    0.649  94.984  -0.274   0.018   0.019   0.964
E N  1DANU                 48    0.574  84.034  -1.924   0.018   0.019   0.964
E T  1DANU                 49    0.554  76.840  -0.044   0.021   0.279   0.699
E F  1DANU                 50    0.425  85.318  -0.043   0.004   0.616   0.381
B L  1DANU                 51    0.167  30.541   1.147   0.023   0.655   0.322
E S  1DANU                 52    0.269  31.550   0.128   0.023   0.655   0.322
B L  1DANU                 53    0.100  18.310  -0.653   0.022   0.552   0.426
E R  1DANU                 54    0.398  91.188  -0.030   0.023   0.655   0.322
B D  1DANU                 55    0.216  31.082  -1.003   0.022   0.552   0.426
B V  1DANU                 56    0.203  31.170  -0.053   0.022   0.552   0.426
B F  1DANU                 57    0.238  47.827  -0.478   0.022   0.359   0.619
E G  1DANU                 58    0.523  41.129  -0.502   0.004   0.085   0.910
E K  1DANU                 59    0.490 100.896  -0.335   0.005   0.045   0.951
E D  1DANU                 60    0.571  82.324   0.377   0.004   0.197   0.799
B L  1DANU                 61    0.072  13.256   0.090   0.003   0.718   0.279
E I  1DANU                 62    0.259  47.841   0.921   0.001   0.900   0.099
B Y  1DANU                 63    0.060  12.929   0.677   0.001   0.959   0.040
B T  1DANU                 64    0.211  29.210   1.463   0.001   0.959   0.040
B L  1DANU                 65    0.028   5.145   0.926   0.000   0.983   0.017
B Y  1DANU                 66    0.103  22.054   1.165   0.000   0.983   0.017
B Y  1DANU                 67    0.065  13.933   0.275   0.000   0.983   0.017
B W  1DANU                 68    0.166  40.043   0.681   0.001   0.959   0.040
E S  1DANU                 69    0.421  49.318   0.536   0.003   0.718   0.279
E G  1DANU                 70    0.579  45.536  -0.758   0.005   0.045   0.951
E K  1DANU                 71    0.581 119.491  -0.238   0.005   0.045   0.951
E K  1DANU                 72    0.602 123.914   0.467   0.019   0.141   0.840
E T  1DANU                 73    0.683  94.663   0.396   0.019   0.141   0.840
E A  1DANU                 74    0.490  54.053   0.582   0.022   0.359   0.619
E K  1DANU                 75    0.508 104.496   0.795   0.021   0.756   0.223
E T  1DANU                 76    0.403  55.910   1.047   0.018   0.846   0.136
E N  1DANU                 77    0.501  73.273   0.091   0.023   0.655   0.322
E T  1DANU                 78    0.362  50.168   0.261   0.069   0.386   0.545
E N  1DANU                 79    0.428  62.615  -0.304   0.148   0.418   0.435
E E  1DANU                 80    0.329  57.546  -0.066   0.169   0.612   0.219
E F  1DANU                 81    0.289  57.942   0.643   0.172   0.690   0.138
B L  1DANU                 82    0.103  18.841   0.475   0.169   0.612   0.219
E I  1DANU                 83    0.457  84.471   0.396   0.148   0.418   0.435
E D  1DANU                 84    0.614  88.549   0.196   0.125   0.227   0.648
B V  1DANU                 85    0.087  13.372   0.274   0.118   0.150   0.732
E D  1DANU                 86    0.491  70.782   0.891   0.053   0.043   0.903
E K  1DANU                 87    0.565 116.303   0.374   0.018   0.019   0.964
E G  1DANU                 88    0.458  36.076  -0.398   0.018   0.019   0.964
B E  1DANU                 89    0.216  37.700   0.302   0.019   0.141   0.840
B N  1DANU                 90    0.280  41.021  -0.143   0.004   0.420   0.576
B Y  1DANU                 91    0.093  19.917   1.345   0.002   0.816   0.182
B C  1DANU                 92    0.029   4.114   0.480   0.011   0.918   0.071
B F  1DANU                 93    0.036   7.145   0.666   0.001   0.959   0.040
B S  1DANU                 94    0.135  15.810   1.011   0.001   0.959   0.040
B V  1DANU                 95    0.025   3.842   0.866   0.001   0.959   0.040
B Q  1DANU                 96    0.120  21.414   0.664   0.001   0.959   0.040
B A  1DANU                 97    0.031   3.372  -0.644   0.001   0.959   0.040
B V  1DANU                 98    0.102  15.739   0.697   0.001   0.900   0.099
B I  1DANU                 99    0.074  13.616   0.438   0.003   0.718   0.279
B P  1DANU                100    0.156  22.136  -0.422   0.022   0.359   0.619
E S  1DANU                101    0.305  35.805  -0.545   0.019   0.141   0.840
E R  1DANU                102    0.448 102.523  -0.181   0.052   0.084   0.864
E T  1DANU                103    0.535  74.205  -0.921   0.184   0.043   0.773
E V  1DANU                104    0.417  64.139  -1.074   0.184   0.043   0.773
B N  1DANU                105    0.300  43.920  -1.715   0.115   0.016   0.868
E R  1DANU                106    0.431  98.699  -0.338   0.058   0.017   0.925
E K  1DANU                107    0.598 123.070  -0.550   0.018   0.019   0.964
B S  1DANU                108    0.246  28.773  -1.376   0.018   0.019   0.964
E T  1DANU                109    0.670  92.874  -0.724   0.018   0.019   0.964
E D  1DANU                110    0.500  72.064  -0.789   0.018   0.019   0.964
B S  1DANU                111    0.212  24.882  -0.764   0.018   0.019   0.964
E P  1DANU                112    0.589  83.593   1.168   0.018   0.088   0.893
E V  1DANU                113    0.443  68.074   0.475   0.021   0.451   0.528
E E  1DANU                114    0.436  76.152   1.513   0.022   0.552   0.426
B C  1DANU                115    0.149  20.990  -0.451   0.021   0.451   0.528
E M  1DANU                116    0.662 132.486  -0.789   0.005   0.015   0.979

Explain the output.  Go back.
```

---
